# Comparative study of bluetongue virus serotype 8 production on BHK-21 cells in a 50L Biostat^®^ STR single-use bioreactors vs roller bottles

**DOI:** 10.1186/1753-6561-7-S6-P83

**Published:** 2013-12-04

**Authors:** Lídia Garcia, Mercedes Mouriño, Alicia Urniza

**Affiliations:** 1 Zoetis Manufacturing & Research Spain, S.L Pfizer Olot S.L.U., Ctra. Camprodon s/n, La Riba, 17813 Vall de Bianya (Girona), Spain

## Background

Bluetongue is a major disease of ruminant livestock that can have a substantial impact on income and animal welfare. Bluetongue virus serotype 8 (BTV-8) first emerged in the European Union in 2006, peaking at 45,000 cases in 2008. Zoetis (formerly Pfizer Animal Health) licensed bluetongue vaccines (Zulvac 4 Ovis, Zulvac 1 Ovis, Zulvac 1 Bovis, Zulvac 8 Ovis and Zulvac 8 Bovis and combinations) able to prevent viremia, stressing the role of the vaccine as an aid for the epidemiological control of the disease.

One important issue to be taken into account in the development of vaccines is their cost, especially in veterinary use. Vaccine production requires high-yield, stable bioproduction systems and implementation of new technologies.

Mammalian cells are the substrate for production of most of the veterinary vaccines. BHK-21 cells are commonly used to produce bluetongue vaccines.

As an example, the use of the BTV-8 vaccine is routinely produced in roller bottles (RB). The aim of this study is to investigate Single-Use Bioreactor technology as an alternative to RB. This technology combines the basic concept of allowing the cells to attach to a surface (microcarriers) with the advantages of suspension, which allows a better control of culture conditions and systematic and automatic culture process.

Single use technology can also be an alternative to conventional production methods reducing facility complexity, possibility of the rapid expansion of the capacity of the production and to avoid the cleaning process and reduction of the risk of cross-contamination. Lower culture handling and more homogeneity can be achieved.

Selection of appropriate culture conditions can be important to achieve consistent cell culture and virus production across sites and scales. Because characteristics like tank geometry and hardware (impellers, sparger) are not subject to change during scale-up, the scalability from 50L to 1000L in the BIOSTAT^®^ STR bioreactor can be an easy strategy for our production process.

## Materials and methods

### Cell line

BHK-21. These cells were used because they are permissible to BTV replication. All cells were cultured at 37°C in MEM-G medium supplemented with serum.

### Virus strain

BTV-8, strain BEL2006/02, supplied by "Veterinary and Agrochemical Research Centre" (VAR-CODA-CERVA), Ukkel, Belgium.

### Cultivation system

The growth of the BHK-21 cells and production of virus was performed in roller bottles and 50L single-use bioreactor BIOSTAT^®^ STR (Sartorius Stedim Biotech).

BHK-21 cells were grown in microcarriers Cytodex-3 at 3g/L into the STR bioreactor and the cell production was optimized with respect to pH, temperature, stirring speed and aeration rate.

Viable cell number was evaluated using the crystal violet dye nucleus staining method.

Virus infection and titration.

The virus chosen to compare and prove the suitability of Single use technology for the production of viral vaccines was BTV-8.

Confluent cells were infected at a constant MOI and harvesting was done at 100% CPE.

Virus production was calculated according to the Spearman-Kärber method, expressing the result in tissue culture infectious doses (50%) (TCID_50_).

Cell growth and BTV-8 antigen production in the BIOSTAT^® ^STR bioreactor was conducted at the optimal conditions determined previously on conventional bioreactors.

### Microcarriers elimination

Taking into account that for vaccine formulation microcarriers must be eliminated from the viral suspension, filtration through Sartopure PP2 cartridges (from Sartorius Stedim Biotech) was performed.

## Results

The final goal is to maximize productivity preserving its quality.

How? *By increasing cell concentration and cell productivity*.

To demonstrate the feasibility of bioreactors for microcarriers cell cultures, the growth of BHK-21 cells in roller bottles, and in the BIOSTAT^® ^STR bioreactor was evaluated and compared.

**Figure 1:**
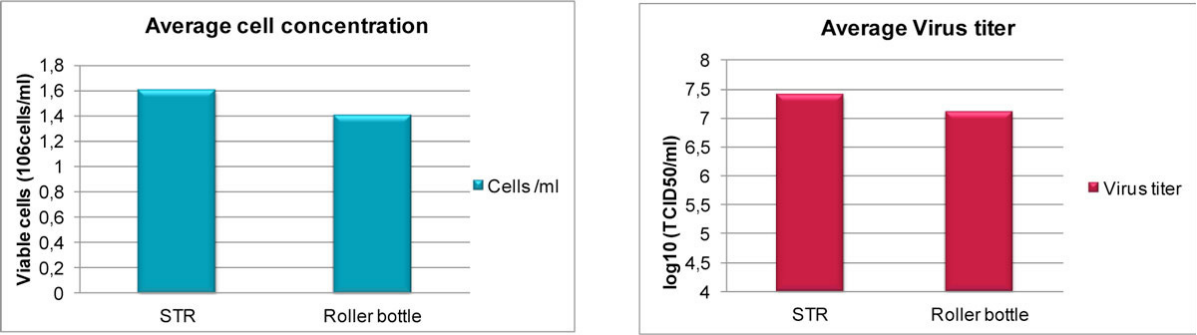
Comparison of cell growth and virus titer in roller bottles and in 50 liter BIOSTAT^®^CultiBagSTR single-use bioreactor

Results prove that when using the 50L BIOSTAT^® ^STR bioreactor, BHK-21 cells are attached and grow efficiently on microcarriers. Cell concentration yield in terms of average was higher than in roller bottles (Figure 1).

The virus titers reached in the BIOSTAT^®^ STR bioreactor were equal o higher than the levels obtained in roller bottles (Figure1).

## Conclusions

▪ Comparable results between Roller bottles and 50L BIOSTAT^®^ STR bioreactor

✓ cell density

✓ productivity

✓ product quality

▪ BHK-21 cells grow efficiently on microcarriers. Conditions for cell attachment in terms of mixing conditions were optimized.

▪ BTV-8 antigen with satisfactory yields can be obtained by culturing BHK-21 in a 50L BIOSTAT^®^ STR bioreactor.

▪ As expected, high density of BHK-21 cultures showed increased productivity.

▪ Microcarrier filtration causes no significant drop in virus titer.

▪ With the conditions established with the 50L BIOSTAT^®^ STR bioreactor the reproducibility and the scale-up from 50L to 1000L can be easily performed.

▪ Single-Use Bioreactor technology is a good alternative to Roller Bottles and is a suitable system for propagation of BTV-8 virus using adherent BHK cells on microcarriers. Involving reduction of costs, cleaning, sterilization etc.

